# A randomized controlled trial to evaluate the role of interferon as initial and maintenance therapy in patients with follicular lymphoma

**DOI:** 10.1054/bjoc.2001.1822

**Published:** 2001-07

**Authors:** A Rohatiner, J Radford, D Deakin, H Earl, S B Love, O Price, A Wilson, T A Lister

**Affiliations:** ICRF Medical Oncology Unit, St. Bartholomew’s Hospital, London, West Smithfield, EC1A 7BE; Christie CRC Research Centre, Christie Hospital, Wilmslow Road, Manchester, M20 9BX; Birmingham Oncology Centre, Queen Elizabeth Hospital, Dudley Road, Birmingham, B18 7QH; ICRF Medical Statistics Group, Institute of Health Sciences, Old Road, Oxford, Headington OX3 7LF

**Keywords:** interferon, initial therapy, maintenance, follicular lymphoma

## Abstract

The purpose of this study was to evaluate the role of interferon as initial and maintenance therapy in patients with newly diagnosed follicular lymphoma. Between 1984 and 1994, 204 patients with newly diagnosed Stage III or Stage IV follicular lymphoma were randomized to receive either, Chlorambucil (CB): 10 mg daily for 6 weeks, followed by a 2-week interval, with 3 subsequent 2-week treatment periods at the same dose, separated by 2-week intervals, or, CB given concurrently with interferon (IFN). IFN was given at a dose of 3 × 10^6^units thrice weekly, subcutaneously, throughout the 18-week treatment period. Responding patients were subsequently randomized to receive maintenance IFN at the dose and schedule described above, or to expectant management. The overall response rate was 161/204 (78%), complete remission being achieved in 24% of patients. Neither the addition of IFN to the initial treatment, nor the use of maintenance IFN influenced response rate, remission duration or survival. This study was undertaken to determine whether IFN, given in combination with, and then subsequent to, CB would alter the clinical course of patients with follicular lymphoma. Disappointingly, this objective was not achieved, no advantage having been demonstrated for the addition of IFN. © 2001 Cancer Research Campaign http://www.bjcancer.com


British Journal of Cancer (2001) **85**(1), 29–35
© 2001 Cancer Research Campaign
doi: 10.1054/ bjoc.2001.1822, available online at http://www.idealibrary.com on IDEAL®

http://www.bjcancer.com

### A randomized controlled trial to evaluate the role of interferon as initial and maintenance therapy in patients with follicular lymphoma

**A Rohatiner[1], J Radford[2], D Deakin[2], H Earl[3], SB Love[4], O Price[1], A Wilson[1] and TA Lister[1]**

[1]ICRF Medical Oncology Unit, St. Bartholomew's Hospital, West Smithfield, London EC1A 7BE; [2]Christie CRC Research Centre, Christie Hospital, Wilmslow Road, Manchester M20 9BX; [3]Birmingham Oncology Centre, Queen Elizabeth Hospital, Dudley Road, Birmingham B18 7QH; [4]ICRF Medical Statistics Group, Institute of Health Sciences, Old Road, Headington, Oxford OX3 7LF

**Summary** The purpose of this study was to evaluate the role of interferon as initial and maintenance therapy in patients with newly diagnosed follicular lymphoma. Between 1984 and 1994, 204 patients with newly diagnosed Stage III or Stage IV follicular lymphoma were randomized to receive either, Chlorambucil (CB): 10 mg daily for 6 weeks, followed by a 2-week interval, with 3 subsequent 2-week treatment periods at the same dose, separated by 2-week intervals, or, CB given concurrently with interferon (IFN). IFN was given at a dose of $3 \times 10^6$ units thrice weekly, subcutaneously, throughout the 18-week treatment period. Responding patients were subsequently randomized to receive maintenance IFN at the dose and schedule described above, or to expectant management. The overall response rate was 161/204 (78%), complete remission being achieved in 24% of patients. Neither the addition of IFN to the initial treatment, nor the use of maintenance IFN influenced response rate, remission duration or survival. This study was undertaken to determine whether IFN, given in combination with, and then subsequent to, CB would alter the clinical course of patients with follicular lymphoma. Disappointingly, this objective was not achieved, no advantage having been demonstrated for the addition of IFN. © 2001 Cancer Research Campaign http://www.bjcancer.com

**Keywords**: interferon; initial therapy; maintenance; follicular lymphoma

Interferon (IFN) was introduced into the treatment of follicular lymphoma almost 20 years ago, on the basis of interesting data in L1210 leukaemia and AKR lymphoma (Gresser et al, 1970, 1976) but with limited understanding of its mode of action. Phase II studies, using an empirical dose and schedule derived from the original trial in osteogenic sarcoma (Strander et al, 1979) showed a response rate of 30–50%, regardless of the source of interferon (Gutterman et al, 1980; Louie et al, 1981; Foon et al, 1984; Quesada et al, 1984; Horning et al, 1985; O'Connell et al, 1986; Wagstaff et al, 1986; Leavitt et al, 1987). The remissions were virtually always incomplete and often took several months to achieve. Further pre-clinical data suggested synergy between interferon and conventional cytotoxic drugs (Chirigos and Pearson, 1973; Gresser et al, 1978; Balkwill and Moodie, 1984) and the latter observations formed the rationale for evaluating the combination of IFN and Chlorambucil (CB) in patients with low-grade lymphoma. Feasibility was demonstrated in patients with recurrent or refractory disease and responses were observed in patients deemed to be refractory to CB alone (Chisesi et al, 1987; Rohatiner et al, 1987). A randomized study was therefore designed to evaluate the use of IFN in 2 settings: in addition to CB as initial therapy, and as maintenance therapy in newly diagnosed patients with advanced disease. The results form the basis of this report.

*Received 1 December 2000*
*Revised 4 December 2000*
*Accepted 9 February 2001*

*Correspondence to:* AZS Rohatiner

### PATIENTS AND METHODS

### Patients

231 newly diagnosed patients with Stage III or IV follicular lymphoma whose clinical characteristics at presentation are shown in Table 1 were entered into the study between October 1984 and October 1994. Patients were treated at 3 main centres: the Christie Hospital, Manchester (104 patients), St. Bartholomew's Hospital, London (60 patients), and Queen Elizabeth Hospital, Birmingham (22 patients). 18 patients were referred from other hospitals. 204 patients form the basis of this analysis, 27 having been excluded for the following reasons: incorrect histology on review: 14, incorrect stage on review: 10, previous treatment: 3.

Stage had been determined from the history, accompanied by clinical examination, computed axial tomography (CT) of the chest, abdomen and pelvis, and unilateral iliac crest bone marrow aspirate and biopsy. Liver involvement was diagnosed on the basis of confirmation of defects seen on CT scanning by ultrasonography.

### Treatment

The overall strategy is outlined in Figure 1. After informed consent had been obtained, patients were randomly allocated to receive either, CB: 10 mg daily for 6 weeks, followed by a 2-week interval, with 3 subsequent 2-week treatment periods at the same dose, separated by 2-week intervals or the latter given concurrently with IFN. Patients randomized to CB + IFN, received the latter at a dose of $3 \times 10^6$ units thrice weekly, subcutaneously,

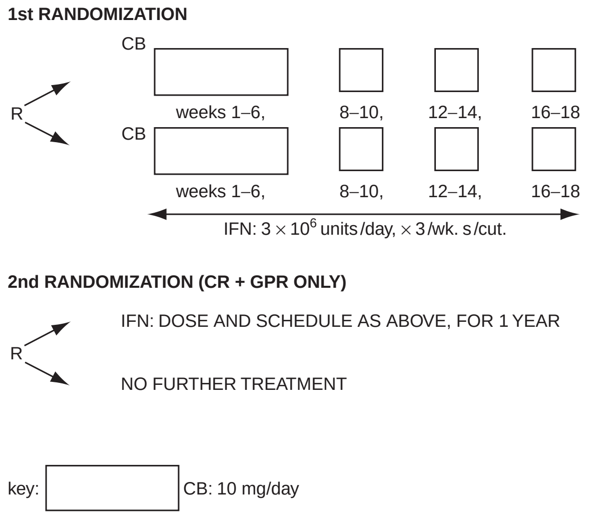

**Figure 1** Treatment strategy

**Table 1** Clinical features at presentation

| | CB | CB + IFN | Total |
|---|---|---|---|
| M:F | 49:51 | 62:42 | 111:93 |
| Age median (range) | 53 (29–78) | 51 (25–81) | 52 (25–81) |
| Stage IIIA | 17 | 17 | 34 |
| IIIB | 8 | 6 | 14 |
| IVA | 42 | 47 | 89 |
| IVB | 33 | 34 | 67 |
| Hepatomegaly | 14 | 21 | 35 |
| Splenomegaly | 36 | 36 | 72 |

*This study was conducted at hospitals that used the Kiel classification. No differentiation has therefore been made between histological subsets of follicular lymphoma.

concurrently throughout the 18-week treatment period. 100 patients were randomized to CB alone, 104 to the combination.

CB or CB + IFN was discontinued for 2 weeks in the first instance in patients with treatment-induced neutropenia (neutrophils $< 1 \times 10^9\ l^{-1}$) or thrombocytopenia (platelets $< 100 \times 10^9\ l^{-1}$) and restarted at full dosage upon recovery. Persistent or recurrent cytopenia led to a 50% reduction in the CB dose, or ultimately to discontinuation of therapy. Intolerable subjective side-effects attributable to IFN were managed in the first instance by a 50% dose reduction and if they persisted, by discontinuation.

For the first 2 years of the study, patients with responding or ‘stable’ disease were randomized to maintenance IFN or to no further treatment, following stratification for response. Subsequently, randomization was limited to patients in whom a complete or ‘good partial response’ (GPR) was achieved (see below), it being considered more appropriate to administer alternative treatment to those in whom less than GPR was achieved. Maintenance IFN (at the same dose and schedule as used initially) was given for one year, except at the Christie Hospital, where it was stopped after 6 months. Outcome (in terms of remission duration and survival) was the same, irrespective of the length of time for which maintenance IFN was given, the results have therefore been combined.

**Table 2** Numbers of patients at each randomization

| Initial therapy | 2nd randomization | |
|---|---|---|
| | IFN | NFT |
| CB | 33 | 29 |
| CB + IFN | 27 | 19 |
| Total | 60 | 48 |

NFT = No further treatment.

108 of 126 eligible patients were randomized, 18 were not, for the following reasons: error; 6, prior IFN toxicity; 5. 4 patients declined randomization, and 1 developed recurrent lymphoma within 4 weeks of finishing initial treatment. In 2 patients, second randomization was considered inappropriate (due to persistent neutropenia, and the development of angina respectively). The number of patients actually receiving CB or the combination, followed by IFN or no further treatment is shown in Table 2.

Patients were seen every 2 weeks whilst receiving Chlorambucil and monthly whilst receiving maintenance IFN (or being managed expectantly if randomized to this arm of the study). Subsequently, all patients were seen at 3 monthly intervals. Management following recurrence was determined by the circumstances: in younger patients, further chemotherapy was given to induce second remission with a view to proceeding to myeloablative therapy supported by autologous bone marrow transplantation (Rohatiner et al, 1994).

### Post-treatment evaluation and definition of response

Formal re-evaluation comprising clinical examination, CT scanning and repeat bone marrow biopsy (if previously positive) was undertaken one month after completion of initial treatment (unless there was a clinical indication to do so earlier).

Response was defined as either: complete remission (CR): no evidence of residual disease; good partial remission (GPR): clinical complete response with minimal residual abnormality on CT scans or bone marrow trephine; poor partial remission (PPR): >50% reduction in any measurable lesion associated with improvement in nonmeasurable involvement, i.e. less than GPR; failure to respond: anything less than PPR.

### Statistical analyses

In the original study protocol, it was considered necessary to accrue 200 patients in order to demonstrate a difference in remission duration of 35–40%. The following factors were tested for possible influence on response, remission duration and survival: gender, age, presence of B symptoms, hepatosplenomegaly, stage, anaemia (Hb < 11.5 g), abnormalities of liver function, performance status and treatment with IFN.

### *Randomization balance*

The study was randomized to achieve balance between the treatment groups for both known and unknown prognostic factors. Each variable (other than age) was therefore considered against the randomization code. Balance for age (the only continuous variable) was considered by looking at the median for each randomization group.

 © 2001 Cancer Research Campaign

*Predicting response*

Variables were considered one at a time by calculating Fisher's exact test on tables of the variable vs response. Variables found to be significant at $P < 0.1$ were put into a logistic model, together with liver function (to adjust for balance). Only liver function and variables with $P < 0.05$ were retained. Results of the logistic regression analysis are given in terms of Odds ratios, an Odds ratio of 2 for Hb (normal vs low) being interpreted as a patient with a normal Hb having twice the chance of response as a patient with a low Hb, all other prognostic factors being the same.

*Remission duration analysis*

Remission duration was defined as the time from date of response to date of recurrence and was considered only for those patients in whom CR or GPR was achieved. Univariate analysis was performed by means of the Log-rank test and survival plots drawn using the Kaplan–Meier method (Kaplan and Meier, 1958). All variables significant at $P < 0.1$ in the log-rank analysis were put into a backward stepwise Cox Regression model (Kaplan and Meier, 1958). The proportional hazards model assumption was assessed by means of log minus log hazard plots. The Cox results are expressed in terms of hazard ratios, a hazard ratio of 2 for anaemia again being interpreted as a patient with anaemia having twice the risk of recurrence as a patient with a higher Hb, all other prognostic factors being the same.

*Survival*

Survival was defined as the time from first randomization until death, or last follow-up. Analyses were performed using the same methods as described above for remission duration.

### RESULTS

### Response (Table 3)

The overall response rate was 78% (161/204), CR being achieved in 24% of patients (49/204). For patients who received CB as initial treatment, the overall response rate was 84% (84/100), for those receiving CB + IFN it was 74% (77/104). However, the CR + GPR rate was higher in patients receiving Chlorambucil alone (70/100, 70% vs 56/104, 54%, $P = 0.02$).

Univariate analysis using the Fisher Exact test showed gender, B symptoms, performance status, liver function, anaemia, age and treatment to predict for response. The final logistic (multivariate) model on 183 patients with complete data is shown in Table 4. 'Forcing' the first randomization code into this model, the odds ratio for CB + IFN vs CB alone is 0.5 (with a 95% confidence interval of 0.2–1.1, $P = 0.1$). The addition of IFN to CB as initial treatment did not therefore improve response rate.

**Table 3** Outcome of initial therapy

| | CB | CB + IFN | TOTAL |
|---|---|---|---|
| CR | 28 (28%) | 21 (20%) | 49 (24%) |
| GPR | 42 (42%) | 35 (34%) | 77 (38%) |
| PPR | 14 (14%) | 21 (20%) | 35 (17%) |
| Treatment failed | 16 (16%) | 27 (26%) | 43 (21%) |
| Total | 100 | 104 | 204 |

**Table 4** Prognostic factors for response

| Variable value | Coding | OR | (95% CI) | *P* |
|---|---|---|---|---|
| Gender | F vs M | 2.3 | (1.0–5.2) | 0.04 |
| Liver function | poor vs normal | 0.4 | (0.2–0.95) | 0.04 |
| Performance status | change of one level | 0.5 | (0.3–0.9) | 0.03 |
| Age | 10-year difference | 0.7 | (0.5–1.0) | 0.05 |

OR = odds ratio; CI = confidence interval.

### Duration of remission

Since for most of the duration of the study, only patients in whom CR or GPR was achieved (the majority) continued in the study (the rest receiving alternative therapy), only this sub-group has been included in the analysis of remission duration. With a median follow-up of 8.5 years, the median remission duration is 3.8 years (Figure 2); 88 patients have developed recurrent lymphoma. 2 patients died in remission, the latter have therefore been censored.

On univariate analysis, lymph node enlargement, anaemia and the addition of IFN were significant prognostic factors (liver function being included for adjustment). When liver function was included in the Cox model, no variable was found to be significant (Table 5). 'Forcing' liver function and the addition of IFN into the Cox model (on 116 patients with 78 recurrences) gives a hazard ratio for CB + IFN vs CB of 0.7 (95% confidence interval 0.4–1.1, $P = 0.09$). On multivariate analysis, adjusting for potentially unbalanced factors and for other prognostic factors, the addition of IFN to CB as the initial treatment did not significantly influence remission duration.

### Survival

The median survival was 8.5 years (Figure 2); 93 patients have died, 70 as a consequence of disease progression, 10 of the latter dying of complications of further treatment. 12 patients died of

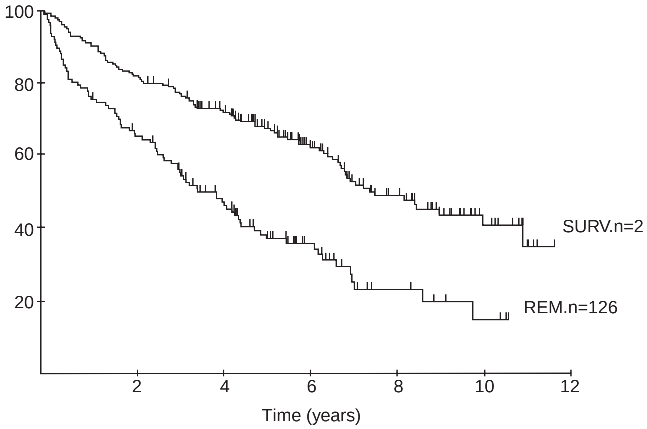

**Figure 2** Survival and duration of remission for all patients

**Table 5** Prognostic factors for remission duration

| Variable | Coding | HR | (95% CI) | *P* value |
|---|---|---|---|---|
| Liver function | poor vs normal | 1.4 | (0.8–2.2) | 0.2 |
| 1st. randomization | CB + IFN vs CB | 0.76 | (0.4–1.1) | 0.09 |

HR = hazard ratio; CI = confidence interval.

*© 2001 Cancer Research Campaign*

causes unrelated to lymphoma or its treatment (myocardial infarction 4, other malignancies 4, cerebrovascular accident 2, haemorrhage 1, pulmonary embolism 1).

On univariate analysis, B symptoms, liver function, performance status, splenomegaly, anaemia and age were significant prognostic factors. The final Cox model results (on 183 patients with 82 deaths) are shown in Table 6. 'Forcing' the addition of IFN into this model gives a hazard ratio for CB + IFN vs CB of 1.00, (95% confidence interval 0.64–1.6, $P$ = 1.0). On multivariate analysis, adjusting for potentially unbalanced factors and for other prognostic factors, the addition of IFN to CB as initial treatment did not influence survival.

### Effect of maintenance IFN on remission duration

Complete or good partial remission was achieved in 126 patients who were therefore eligible for second randomization. 108/126 were actually randomized, 60 to receive maintenance IFN, 48 to no further treatment. Overall, 74 patients developed recurrent lymphoma; 2 who died without recurrence are censored.

On univariate analysis, none of the factors considered were significant. On multivariate analysis, 'forcing' the use of maintenance IFN into a Cox model gives a hazard ratio (for no further treatment vs IFN maintenance) of 1.4, (95% confidence interval 0.9–2.2, $P$ = 0.1). The use of maintenance IFN did not therefore significantly influence remission duration. Considering the 4 possible treatment combinations resulting from the first and second randomizations, (analysing 108 patients with 74 recurrences in a Cox model) no sequence of treatments was significantly better in terms of remission duration than CB followed by no further treatment. These results are shown in full in Table 7 and in Figure 3.

**Table 6** Prognostic factors for survival

| Variable | Coding | HR | (95% CI) | *P* value |
|---|---|---|---|---|
| Liver function | poor vs normal | 1.9 | (1.2–3.0) | 0.005 |
| Performance status | change of one level | 1.6 | (1.2–2.3) | 0.005 |
| Age | 10-year difference | 1.5 | (1.2–1.9) | <0.001 |

HR = hazard ratio; CI = confidence interval.

**Table 7** Cox model results for 4 treatment combinations in terms of remission duration

| Treatment<br>value | HR | (95% CI) | *P* |
|---|---|---|---|
| CB + IFN → NFT vs CB → NFT | 0.8 | (0.4–1.5) | 0.4 |
| CB → IFN vs CB → NFT | 0.7 | (0.4–1.3) | 0.2 |
| CB + IFN → IFN vs CB → NFT | 0.6 | (0.3–1.1) | 0.09 |

NFT = no further treatment; HR = hazard ratio; CI = confidence interval.

**Table 8** Cox model results for 4 treatment combinations in terms of survival

| Variable | Coding | HR | (95% CI) | *P* value |
|---|---|---|---|---|
| Age | 10-year difference | 1.6 | (1.2–2.2) | 0.003 |
| Treatment | CB + IFN → NFT vs CB → NFT | 1.5 | (0.6–3.9) | 0.4 |
| | CB → IFN vs CB → NFT | 0.8 | (0.3–1.9) | 0.6 |
| | CB + IFN → IFN vs CB → NFT | 0.8 | (0.3–2.2) | 0.7 |

NFT = no further treatment; HR = hazard ratio; CI = confidence interval.

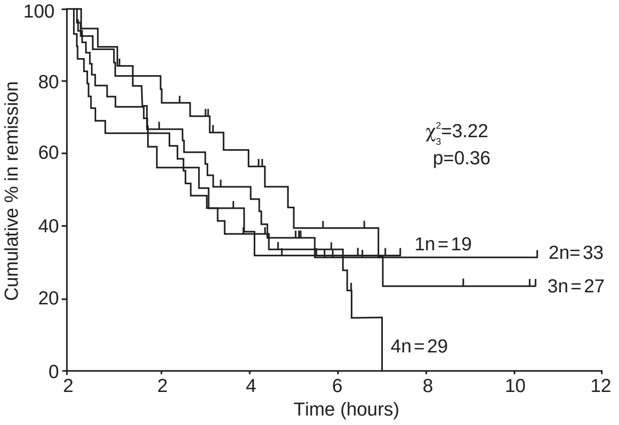

**Figure 3** Remission duration according to treatment: (1) CB + IFN → NFT; (2) CB→ IFN; (3) CB + IFN → IFN; (4) CB → NFT

### Effect of maintenance IFN on survival

Once more, only patients in whom CR or GPR was achieved were considered. Survival from second randomization was analysed using the same methods as described above for analysis of survival from the time of diagnosis. On univariate analysis, liver function, anaemia and age were found to be significant prognostic factors. On multivariate analysis, (using the Cox model on 108 patients with 34 deaths), only age gave a hazard ratio of 1.6 (confidence interval 1.2–2.2, $P$ = 0.003). 'Forcing' the second randomization into this model gives a hazard ratio for IFN maintenance vs no further treatment of 1.5 (95% confidence interval 0.7–2.9, $P$ = 0.3). The use of maintenance IFN did not therefore significantly influence survival. Considering all 4 treatment combinations in the Cox model, no combination was significantly better in terms of survival than standard treatment with CB followed by no further treatment (Table 8). Survival curves for the 4 patient groups are shown in Figure 4.

### Toxicity

7 patients had to discontinue CB due to clinical toxicity. All subsequently developed recurrent lymphoma; 4 are well, 3 died of

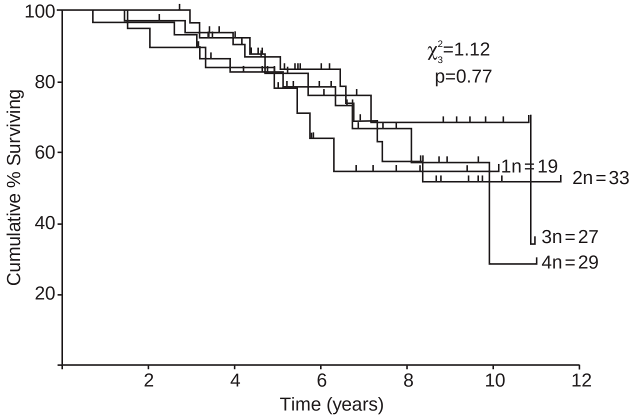

**Figure 4** Survival according to treatment: (1) CB + IFN → NFT; (2) CB → IFN; (3) CB + IFN → IFN; (4) CB → NFT

*© 2001 Cancer Research Campaign*

progressive disease. Subjective Interferon toxicity with the *initial* treatment prevented continuation of the drug in 9 patients. 6 of the latter remain alive, 3/6 having been treated for recurrent lymphoma, 3 patients have died of progressive disease.

Haematological toxicity, resulting in an interruption in treatment or dose modification was significantly greater in patients receiving the combination (11/100: CB alone vs 37/104 for CB + IFN, $P < 0.001$). In addition, 5 patients experienced haematological toxicity with maintenance IFN necessitating interruption of treatment.

### DISCUSSION

This study was undertaken to determine whether IFN, given initially in combination with, and then subsequent to, Chlorambucil, would alter the clinical course of patients with follicular lymphoma. Disappointingly, this objective was not achieved, no significant advantage being demonstrated for the addition of IFN to initial treatment or as maintenance therapy. An interim analysis had shown a significant difference in remission duration in favour of maintenance IFN (Price et al, 1991), however, with longer follow-up, this difference has been abrogated.

With regard to improving response rate, in this study, the addition of IFN not only did not help, but was associated with a lower response rate. The explanation for this may be the greater degree of haematological toxicity incurred with the combination, which in turn resulted in delays in administering Chlorambucil. Neither of the 2 other published studies in which IFN has been combined with an alkylating agent has in fact shown any advantage for the combination (Chisesi et al, 1991; Peterson et al, 1997). In contrast, the study reported by the 'GELF' Group (Solal-Celigny et al, 1993), did show a significantly higher response rate for IFN given with a more intensive, Adriamycin-containing regimen.

The rationale for adding IFN to alkylating agent therapy was based on 2 murine studies. Early work in AKR mice had demonstrated an 'additive' effect with a 200% increase in survival for mice treated with the combination of IFN and Cyclophosphamide (Gresser et al, 1978). A subsequent study (using the same combination of drugs) in a breast cancer xenograft growing in nude mice confirmed a synergistic response (Balkwill and Moodie, 1984). The latter study also indicated that the antitumor effect was greatest when the 2 drugs were used concurrently rather than sequentially.

The precise mechanisms of action of IFN in follicular lymphoma are unclear but probably represent a direct anti-proliferative effect (Balkwill and Taylor-Papdimitriou, 1978; Balkwill et al, 1978, Taylor-Papdimitriou, 1980). It is, however, possible that indirect effects on drug metabolism (Friedman et al, 1979; Singh and Renton, 1981; Marguett et al, 1983; Stolfi et al, 1983) are also involved.

Although it was not the case in the present study, as mentioned above, the use of IFN as part of initial treatment has been found to prolong remission duration (Smalley et al, 1992; Solal-Celigny et al, 1993; Anderson and Smalley, 1993; Solal-Celigny, 1997; Arranz et al, 1998) and in the 'GELF' study, survival (Solal-Celigny et al, 1993). However, the latter study also had a maintenance phase, it is therefore difficult to separate out the influence of continuing IFN from that of adding IFN to the initial therapy. With regard to prolongation of *survival* in the 'GELF' study, the question as to whether this reflects delay in time to transformation (to large B-cell histology), or a reduction in the incidence of transformation has been addressed; the rate of transformation was the same in the 2 treatment arms (Solal-Celigny, 1997).

Some studies were specifically designed to evaluate the use of maintenance IFN. Neither of the 2 other trials in which IFN maintenance followed treatment with an alkylating agent (Chlorambucil or Cyclophosphamide) show any advantage for maintenance IFN (Chisese et al, 1991; Peterson et al, 1997). However, in a trial conducted by the European Organization for the Research and Treatment of Cancer, in which initial treatment comprised Cyclophosphamide, Vincristine and Prednisolone (CVP, with or without radiotherapy to large nodal masses), there was a trend towards improved time to progression in the IFN–treated group but this did not reach statistical significance (Hagenbeek et al, 1998). This was not the case in a study from Spain (Arranz et al, 1998), in which patients received CVP +/–IFN followed by a second randomization to IFN or to no further treatment. The German Low-grade Lymphoma Study Group trial (Unterhalt et al, 1996) does show a significant prolongation of disease-free survival with maintenance IFN (following initial therapy with either Prednimustine and Mitoxantrone, or CVP). However, this study is different from the rest; there being no fixed time limit for treatment with IFN, the drug being given until recurrence. In contrast, in the present study, there was in fact no difference in outcome between patients treated at the Christie Hospital where maintenance IFN was given only for 6 months and those treated at St Bartholomew's Hospital where IFN was continued for 1 year (data not shown).

A Mexican study also shows both remission duration and survival to be significantly longer in a group of patients randomized to receive IFN after CR had been achieved with 3 sequential regimens followed in most patients by radiotherapy (Aviles et al, 1996). The obvious exception to these positive results is the trial conducted by the South-West Oncology Group (SWOG), in which the use of maintenance IFN given after the intensive, Adriamycin-containing regimen 'PROMACE-MOPP' (and in some patients, involved field radiotherapy) did not influence remission duration or survival (Fisher et al, 2000).

With regard to prognostic factors, the addition of Interferon did not confer benefit in any particular group. (It was not possible to assess the influence of a high LDH level, since LDH was not routinely measured at the time that this study began.) Older age was the only factor that correlated significantly with worse survival, in agreement with most previous analyses (Rudders et al, 1979; Gospodarowicz et al, 1984; Kantarjian et al, 1984; Gallagher et al, 1986; Lawrence et al, 1988; Steward et al, 1988; Lepage et al, 1990; Leonard et al, 1991; Romaguera et al, 1991; Soubeyran et al, 1991).

The discrepancies between the various studies may to some extent be explained by variations in study design and differences in selection criteria. Some trials included patients with low-grade lymphoma, but not necessarily only follicular lymphoma. In some, for example the 'GELF' study (Solal-Celigny et al, 1993), only patients considered to have an adverse prognosis were eligible, whereas in contrast, the ECOG study included patients with 'indolent disease' who were treated at the time of diagnosis, irrespective of whether there was a specific indication for treatment (Smalley et al, 1992).

In general however, the studies with the best results are those in which Interferon has been combined with, or followed, relatively intensive, initial chemotherapy (Smalley et al, 1992; Solal-Celigny

*© 2001 Cancer Research Campaign* 

et al, 1993; Aviles et al, 1996; Unterhalt et al, 1996), the SWOG study clearly being an exception (Fisher et al, 2000). The cumulative dose of IFN may also be important; the 'GELF' (Solal-Celigny et al, 1993), Mexican (Aviles et al, 1996) and German (Unterhalt et al, 1996) studies used a cumulative dose higher than that used in most of the others.

In order to clarify these discrepancies, a meta-analysis of 8 randomized trials has been conducted (Rohatiner et al, 1998). Only patients with follicular lymphoma were considered. No significant advantage was demonstrated for the addition of IFN to initial treatment. The use of IFN as part of initial therapy or as maintenance therapy did significantly improve remission duration ($P$ = 0.001) and survival ($P$ = 0.002) but there was significant heterogeneity between studies. This was clarified when it became apparent that the improvement was only true for studies in which a relatively intensive, Adriamycin (or equivalent) containing initial chemotherapy was used. With regard to 'dose intensity', in studies using $> 36 \times 10^6$ units of IFN per month, or a total cumulative dose $>1000 \times 10^6$ units, the addition of IFN significantly improved survival, but this effect 'lost' significance when the intensity of initial chemotherapy was included in a multivariate regression analysis (Gregory, 1999). Thus, the meta-analysis results confirm the impression that IFN is most effective when used with, or following, more intensive chemotherapy.

These results need to be seen within the context of other current experimental strategies for follicular lymphoma. High-dose treatment with autologous haemopoietic cell support (Rohatiner et al, 1994; Freedman et al, 1997, 1999; Apostolidis et al, 1999, 2000), Fludarabine-containing regimens that may induce 'molecular remission' (McLaughlin et al, 1996; Crawley et al, 2000; Grillo-Lopez et al, 2000), antibody therapy (McLaughlin et al, 1998; Rogers et al, 1996) and targetted irradiation (Press et al, 1995, Kaminski et al, 1996, Vose et al, 2000) are currently being evaluated. It is against this overall background that the place of Interferon must be considered.

### ACKNOWLEDGEMENTS

We are most grateful to the medical and nursing staff involved in the clinical care of these patients and to the referring physicians, especially, Dr J Sweetenham, Professor Barry Hancock and Dr Elizabeth Miller. We thank Schering-Plough for providing the Interferon, Virginia Sykes and Dorothy Brown for their help in collation of data and Chris Sykes and Margaret Cresswell for preparing the manuscript.

### REFERENCES

Andersen JW and Smalley RV (1993) Interferon Alfa plus chemotherapy for non-Hodgkin's lymphoma five-year follow-up. *N Engl J Med* **329**: 1821–1822

Apostolidis J, Foran JM, Johnson PWM, et al. (1999) Patterns of outcome following recurrence after myeloablative treatment with autologous bone marrow transplantation for follicular lymphoma. *J Clin Oncol* **17**: 216

Apostolidis J, Gupta RK, Grenzelias D, et al. (2000) High-dose therapy with autologous bone marrow support as consolidation of remission in follicular lymphoma: long term clinical and molecular follow-up. *J Clin Oncol* **18**: 527

Arranz R, Garcia-Alfonso P, Sobrino P, Zamora P, Carrion R, Garcia-Larana J, Perez G, Lopez J, Lavilla E, Lozano M, Rayon C, Colomer R, Baron MG, Flores E, Perez-Manga G and Fernandez-Ranada JM (1998) Role of Interferon Alfa-2b in the induction and maintenance treatment of low-grade non-Hodgkin's lymphoma: Results from a prospective, multicenter trial with double randomization. *J Clin Oncol* **16**: 1538–1546

Aviles A, Duque G, Talavera A and Guzman R (1996) Interferon Alpha 2b as maintenance therapy in low grade malignant lymphoma improves duration of remission and survival. *Leuk Lymphoma* **20**: 495–499

Balkwill FR and Taylor-Papdimitriou J (1978) Interferon affects both $G_1$ and $G_2$ in cells stimulated from quiescance to growth. *Nature* (Lond) **274**: 800

Balkwill FR and Moodie EM (1984) Positive interactions between human interferon and cyclophosphamide or adriamycin in a human tumor model system. *Cancer Res* **4**: 904–908

Balkwill FR, Watling D and Taylor-Papadimitriou J (1978) Inhibition by lymphoblastoid Interferon of growth cells derived from human breast. *Int J Cancer* 258

Chirigos MA and Pearson JW (1973) Cure of murine leukaemia with drugs and interferon treatment. *J Natl Cancer Inst* **51**: 1367–1368

Chisesi T, Capnist G, Vespignani M and Cetto G (1987) Interferon alfa-2b and chlorambucil in the treatment of non-Hodgkin's lymphoma. *Invest New Drugs* **5**, Suppl: S35–40

Chisesi T, Congiu M, Contu A, Coser P, Moretti C, Porcellini A, Rancan L 6th, Salvagno L, Santini G and Vinante O (1991) Randomized study of Chlorambucil (CB) compared to Interferon (Alfa-2B) combined with CB in low-grade non-Hodgkin's lymphoma: an interim report of a randomized study. Non-Hodgkin's Lymphoma Co-Operative Study Group. *Eur J Cancer* **27** Suppl 4, S31–3

Crawley CR, Foran JM, Gupta RK, et al. (2000) A phase II study to evaluate the combination of fludarabine, mitoxantrone and dexamethasone (FMD) in patients with follicular lymphoma. *Ann Oncol* **11**: 1

Fisher RI, Dana BW, LeBlanc M, Kjeldsberg C, Forman JD, Unger JM, Balcerzak ST, Gaynor ER, Roy V, and Miller T. Interferon alfa consolidation after intensive chemotherapy does not prolong the progression-free survival of patients with low-grade non-Hodgkin's lymphoma: results of the Southwest Oncology Group randomised Phase III study 8809. *J Clin Oncol* 2000, **18**(10): 2010

Foon KA, Sherwin SA, Abrams PA, Longo DL, Fer MF, Stevenson HC, Ochs JJ, Bottino GC, Schoenberger CS and Zeffren J, et al. (1984) Treatment of advanced non-Hodgkin's lymphoma with recombinant leukocyte A interferon. *N Engl J Med* **311**: 1148–1152

Freedman A, Gribben J, Neuberg D, Mauch P, Soiffer R, Anderson K, Fisher D, Schlossman R, Kroon M, Ritz J and Nadler L (1997) Long-term prolongation of disease-free and overall survival following autologous bone marrow transplantation in patients with advanced relapsed follicular lymphoma. *Proc Am Soc Clin Oncol* **16**: 89a

Freedom AR, Neuberg D, Mauch P, et al. (1999) Long-term follow-up of autologous bone marrow transplantation in patients with relapsed follicular lymphoma. *Blood* **94**: 3325

Friedman OM, Myles A and Colvin M (1979) Cyclophosphamide and raised p;hosphoramide mustards. In: I Rosowsky (ed), *Adv in Cancer Chemo* 143

Gallagher CJ, Gregory WM, Jones AE, Stansfeld AG, Richards MA, Dhaliwal HS, Malpas JS and Lister TA (1986) Follicular lymphoma: Prognostic factors for response and survival. *J Clin Oncol* **4**: 1470–1480

Gospodarowicz MK, Bush RS, Brown TC and Chua T (1984) Prognostic factors in nodular lymphomas: A multivariate analysis based on the Princess Margaret Hospital experience. *Int J Radiat Oncol Biol Phys* **10**: 489–497

Gregory W (1999) Personal communication

Gresser I, Brouty-Boye D, Thomas MT and Macierira-Coelho A (1970) Interferon and cell division. I. Inhibition of the multiplication of mouse leukaemia L1210 cells in vitro by an interferon preparation. *Proc Nat Acad Sci* **66**: 1052–1058

Gresser I, Maury C and Tovey MG (1976) Interferon and murine leukaemia VII. Therapeutic effect of interferon preparations after diagnosis of lymphoma in AKR mice. *Int J Cancer* **7**: 647–651

Gresser I, Maury C and Tovey M (1978) Efficacy of combined interferon cyclophosphamide therapy after diagnosis of lymphoma in AKR mice. *Europ J Cancer* **14**: 97–99

Gutterman JU, Blumenschein GR and Alexanian R (1980) Leukocyte interferon-induced tumour regression in human metastatic breast cancer, multiple myeloma and malignant lymphoma. *Ann Intern Med* **93**: 399–406

Hagenbeek A, Carde P, Somers R and Meerwaldt JH, et al. (1998) Maintenance of remission with human recombinant interferon alpha-2a in patients with stage III and IV low-grade malignant non-Hodgkin's lymphoma. *J Clin Oncol* **16**: 41 (abstract)

Horning SJ, Merigan TC and Krown SE (1985) Human interferon alpha in malignant lymphoma and Hodgkin's disease. *Cancer* **56**: 1305–1310

Kaminski MS, Zasadny KR, Francis IR, Fenner MC, Ross CW, Milik AW, Estes J, Tuck M, Regan D, Fisher S, Glenn SD and Wahl RL (1996) Iodine-131 - Anti-B1 radioimmunotherapy for B-cell lymphoma. *J Clin Oncol* **14**: 1974–1981

© 2001 Cancer Research Campaign

Kantarjian HM, Mclaughlin P, Fuller LM, Dixon DO, Osborne BM and Cabanillas FF (1984) Follicular large cell lymphoma: Analysis and prognostic factors in 62 patients. *J Clin Oncol* **2**: 811–819

Kaplan E and Meier P (1958) Nonparametric estimation from incomplete observations. *Am Stat Assoc J* **53**: 457–480

Lawrence TS, Urba WJ, Steinberg SM, Sundeen JT, Cossman J, Young RC and Glatstein E (1988) Retrospective analysis of stage I and II indolent lymphomas at the National Cancer Institute. *Int J Radiat Oncol Biol Phys* **14**: 417–424

Leavitt RD, Ratanathathorn V, Ozer H, Ultmann JE, Portlock C, Myers JW, Kisner D, Norred S, Spiegel RJ and Bonnem EM (1987) Alfa-2b Interferon in the treatment of Hodgkin's disease and non-Hodgkin's lymphoma. *Semin Onc* **14** (2 suppl 2): 18–23

Leonard RC, Hayward RL, Prescott RJ and Wang J (1991) The identification of discrete prognostic groups in low grade non-Hodgkin's lymphoma. *Ann Oncol* **2**: 655–662

Lepage E, Sebban C, Gisselbrecht C, Coiffier B, Harousseau JC, Byron PA and Boiron M (1990) Treatment of low grade non-Hodgkin's lymphomas: assessment of Doxorubicin in a controlled trial. *Hematol Oncol* **8**: 31–39

Lopez-Guillermo A, Cabanillas F, McLaughlin P et al. (2000) Molecular response assessed by PCR is the most important factor predicting failure-free survival in indolent follicular lymphoma: Update of the MDACC series. *Ann Oncol* **11** (Supp 1): 137

Louie AC, Gallagher JG, Sikora K, Levy R, Rosenberg SA and Merigan TC (1981) Follow up observations on the effect of human leukocyte interferon in non-Hodgkin's lymphoma. *Blood* **58**: 712–718

Marguet RL, Schellekens H, Westbroek DL and Jeakel J (1983) Effect of treatment with Interferon and cyclophosphamide on the growth of a spontaneous liposarcoma in rats. *Int J Cancer* **31**: 223

McLaughlin P, Hagemeister FB, Romaguera JE, et al. (1996) Fludarabine, mitoxantrone and dexamethasone: an effective new regimen for indolent lymphoma trial. *J Clin Oncol* **14**: 1262

O'Connell MJ, Colgan JP, Oken MM, Ritts RE Jr., Kay NE and Itri LM (1986) Clinical trial of recombinant leukocyte A interferon as initial therapy for favourable histology non-Hodgkin's lymphomas and chronic lymphocytic leukaemia. *J Clin Oncol* **4**: 128–136

Peterson BA, Petroni GR, Oken MM, Johnson JL, Barcos M and Cooper MR (1997) Cyclophosphamide versus cyclophosphamide plus interferon alfa-2b in follicular low-grade lymphomas: an intergroup phase III trial (CALGB 8691 and EST 7486). *Proc Am Soc Clin Oncol* **16**: 14a

Press OW, Eary JF, Appelbaum FR, Martin PJ, Nelp WB, Glenn S, Fisher DR, Porter B, Matthews DC, Gooley T and Bernstein ID (1995) Phase II trial of 131-I-B1 (anti-CD20) antibody therapy with autologous stem cell transplantation for relapsed B cell lymphomas. *Lancet* **346**: 336–340

Price C, Rohatiner AZS, Steward W, Blackledge G, Crowther D and Lister TA (1991) Interferon – $a_{2b}$ (IFN-$a_{2b}$) as initial therapy in combination with Chlorambucil (CB) and as maintenance therapy in follicular lymphoma (FL). *Eur J Cancer* **27** Suppl 4: S34–S36

Quesada JR, Hawkins M, Horning S, Alexanian R, Borden E, Merigan T, Adams F and Gutterman JU (1984) Collaborative Phase I–II study of recombinant DNA-produced leukocyte Interferon (Clone A) in metastatic breast cancer, malignant lymphoma and multiple myeloma. *Am J Med* **77**: 427–432

Rogers J, Jackson J, Rosenberg J, Varns C, Leonard J and Grillo-Lopez AJ (1996) Clearance of Bcl-2 t(14; 18) from peripheral blood and bone marrow in patients with relapsed low-grade or follicular lymphoma following single-agent therapy with the chimeric anti-CD20 antibody (MAB) IDEC-C2B8. *Ann of Oncol* **7** (Supp 3): 34

Rohatiner AZS, Richards MA, Barnett MJ, Stansfeld AG and Lister TA (1987) Chlorambucil and interferon for low grade non-Hodgkin's lymphoma. *Br J Cancer* **55**: 225–226

Rohatiner AZS, Johnson PWM, Price CGA, Arnott SJ, Amess JAL, Norton AJ, Dorey E, Adams K, Whelan JS, Matthews J, MacCallum PK, Oza AM and Lister TA (1994) Myeloablative therapy with autologous bone marrow transplantation as consolidation therapy for recurrent follicular lymphoma. *J Clin Oncol* **12**: 1177–1184

Rohatiner AZS, Gregory W, Peterson B, Smalley R, Solal-Celigny P, Hagenbeek A, Bijnens L, Unterhalt M, Chisesi T, Aviles A and Lister TA (1998) A meta-analysis of randomised trials evaluating the role of Interferon as treatment for follicular lymphoma. *Proc Am Soc Clin Oncol* **17**: 4a

Romaguera JE, Mclaughlin P, North L, Dixon D, Silvermintz KB, Garnsey LA Velasquex WS, Hagemeister FB and Cabanillas F (1991) Multivariate analysis of prognostic factors in stage IV follicular low-grade lymphoma: A risk model. *J Clin Oncol* **9**: 762–769

Rudders RA, Kaddis M, Delellis RA and Casey H Jr. (1979) Nodular non-Hodgkin's lymphoma: Factors influencing prognosis and indications for aggressive treatment. *Cancer* **43**: 1643–1651

Singh G and Renton KW (1981) Interferon mediated depression of cytochrome P-450 dependent drug biotransformation. *Mol Pharmacol* **20**: 681

Smalley RV, Andersen JW, Hawkins MJ, Bhide V, O'connell MJ, Oken MM and Borden EC (1992) Interferon alpha combined with cytotoxic chemotherapy for patients with non-Hodgkin's lymphoma. *New Eng J Med* **327**: 1336–1341

Solal-Celigny P (1997) Personal communication.

Solal-Celigny P, Lepage E, Brousse N, Reyes F, Haioun C, Leporrier M, Peuchmaur M, Bosley A, Parlier Y and Brice P et al. (1993) Recombinant interferon alfa-2b combined with a regimen containing doxorubicin in patients with advanced follicular lymphoma. *New Eng J Med* **329**: 1608–1614

Soubeyran P, Eghbali H, Bonichon F, Trojani M, Richaud P and Hoerni B (1991) Low-grade follicular lymphomas: Analysis of prognosis in a series of 281 patients. *Eur J Cancer* **27**: 1606–1613.

Spinolo JA, Cabanillas F, Dixon DO, Khorana SM, Mclaughlin P, Velasquez WS, Hagemeister FB, Redman JR and Swan F Jr. (1992) Therapy of relapsed or refractory low-grade follicular lymphomas: Factors associated with complete remission, survival and time to treatment failure. *Ann Oncol* **3**: 227–232

Steward WP, Crowther D, McWilliam LJ, Jones JM, Deakin DP, Todd ID, Blackledge G, Wagstaff J, Scarffe JH and Harris M (1988) Maintenance chlorambucil after CVP in the management of advanced stage, low-grade histologic type non-Hodgkin's lymphoma: A randomized prospective study with an assessment of prognostic factors. *Cancer* **61**: 441–447

Stolfi RL, Martin DS, Sawyer RC and Spiegelman S (1983) Modulation of 5-fluorouracil-induced toxicity in mice with Interferon or with the Interferon inducer, polyinosinic-polycytidylic acid. *Cancer Res* **43**: 561

Strander H, Adamson U, Aparisi T, Brostrom LA, Cantell K, Einhorn S, Hall K, Ingimarsson S, Nilsonne U and Sodergerg G (1979) Adjuvant interferon treatment of human osteosarcoma. Recent Result *Cancer Res* **68**: 40–44

Taylor-Papdimitriou J (1980) Effects of interferons on cell growth and function. In: I Gresser (ed). *Interferon* **2**: 13–46

Unterhalt M, Hermann R, Koch P, Trumper L, Bodenstein H, Dietzefelbinger H, Landys K, Reub M, Vetter H, Maschmeyer G, Freund M, Neubauer A, Engert A, Stauder R, Herold M, Tiemann M, Parwaresch R, Stein H and Hiddemann W (1996) Long term interferon alpha maintenance prolongs remission duration in advanced low grade lymphomas and is related to the efficacy of initial cytoreductive chemotherapy. *Blood* **88** (Suppl 1): abstract 1801

Vose JM, Wahl RL, Saleh M, et al. (2000) Multicentre Phase II study of Iodine 131 tositumomab for chemotherapy-relapsed/refractory low-grade and transformed low-grade B-cell non-Hodgkin's lymphomas. *J Clin Oncol* **18**: 1316

Wagstaff J, Loynds P and Crowther D (1986) A phase II study of human recombinant DNA-a2 interferon in patients with low-grade non-Hodgkin's lymphoma. *Cancer Chemother Pharmacol* **18**: 54–58

*© 2001 Cancer Research Campaign*
